# Crosstalk‐Free, Stretching‐Insensitive Sensor Based on Arch‐Bridge Architecture for Tactile Mapping with Parallel Addressing Strategy toward Million‐Scale‐Pixels Processing

**DOI:** 10.1002/advs.202101876

**Published:** 2021-09-09

**Authors:** Ruomei Shao, Chunnan Wang, Jingru Zhao, Hang Yang, Shuqing Sun

**Affiliations:** ^1^ Institute of Biopharmaceutical and Healthcare Engineering Shenzhen International Graduate School Tsinghua University Shenzhen 518055 China; ^2^ State Key Laboratory for Strength and Vibration of Mechanical Structures International Center for Applied Mechanics Department of Engineering Mechanics Xi'an Jiaotong University Xi'an 710049 China

**Keywords:** large‐scale touch‐sensing, parallel addressing, strain‐insensitivity, tactile mapping, touchpad

## Abstract

In the field of biomimetic electronics, flexible sensors with both high resolution and large size are attracting a lot of attention. However, attempts to increase the number of sensor pixels have been thwarted by the need for complex inner circuits and the resulting interferences with the output. Technological challenges, such as real‐time spatiotemporal mapping and long‐time reliability, must be resolved for large‐scale sensor matrices. This paper reports a simple and robust sensor with an arch‐bridge architecture (ABA) to address these challenges. The device, which consists of an anti‐icing all‐transparent material system, is fabricated by immobilizing ABA ionic arrays on predefined grooves on the substrate. It systematically integrates ABA structure‐designing, resistance‐position‐sensing, and parallel‐addressing logic, allowing for an improvement of three orders of magnitude in the scanning speed (million‐scale pixels) without logical “diagnose confusion.” In addition, it can withstand 100 000 stretching cycles without functional failure. It is also resistant to interferences from stretching. humidity, wet surfaces, and power lines. The proposed strategy is envisaged to serve as a general solution for high‐density, large‐area tactile sensors in various applications.

## Graphical abstract

A simple and robust touch sensor with an arch‐bridge architecture is reported. It systematically integrates resistance‐based touching mechanism and parallel‐addressing logic, allowing for an improvement of three orders of magnitude in the scanning speed (million‐scale pixels) without logical “diagnose confusion”, and can withstand 100 000 stretching cycling without functional failure.

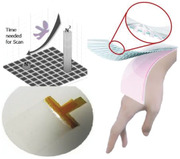

## 1 Introduction

Flexible sensors or touchpads, which exhibit skin‐like properties, offer tactile feedback and serve as an important medium for interactions between humans, robots, and the surroundings.^[^ ^1^, ^2^, ^3^, ^4^, ^5^, ^6^, ^7^, ^8^, ^9^, ^10^ ^]^ Over the past few decades, flexible touch sensors or transducers based on various electrical principles have been explored.^[^ ^3^, ^5^, ^11^, ^12^, ^13^, ^14^, ^15^, ^16^, ^17^, ^18^, ^19^, ^20^ ^]^ However, efforts to improve the sensing abilities of these devices have tended to focus on increasing their pixel density (e.g., making them fingertip like)^[^ ^3^, ^11^, ^12^, ^16^ ^]^ or spatial coverage (e.g., making them skin like).^[^ ^5^, ^10^, ^21^ ^]^ Both approaches lead to a steep growth in the number of pixels required at a quadratic rate and hence long addressing times. As a result, the performance of the sensors developed so far remains unsatisfactory for many applications owing to the poor real‐time spatiotemporal response, reliability, and durability of the large‐scale units.^[^ ^22^, ^23^ ^]^

Large‐scale or high‐resolution sensors consist of millions of units integrated on a common substrate, and even a minor defect will ultimately be amplified. In particular, the failure of the sensor‐to‐sensor connections is a common and dangerous occurrence. In addition, the currently employed scanning strategy, in which the time taken for scanning is directly related to the number of quadratically increasing arrays, is incompatible with high‐pixelated matrices. A high signal‐to‐noise ratio is essential. However, noise arising either from cross‐capacitance between the high‐density units or the strain distribution of the polymer‐based platforms used will result in output signals that are vulnerable to interference from the surroundings. Thus, it is important to develop alternative approaches for fabricating large‐scale sensor matrices that are also robust, exhibit an anti‐interference structure, and allow for real‐time trajectory mapping.

Sensors with a structure that involves fewer electrical interconnections and less‐patterned interlayers would intrinsically be more stretchable if manufactured using bonding elastomers and hyperelastic materials. However, tactile devices based on phenomena such as mutual capacitance, surface capacitance, triboelectricity, and piezoelectricity require an alternating current source and are thus susceptible to cross‐capacitance and strain interference. Resistance‐based touch sensing devices, which can be powered by a direct current source, are more likely to exhibit cross‐talk‐ and strain‐interference‐free functioning and result in highly linear feedback.^[^ ^24^ ^]^ Stretchable multitouch arrays have not yet been realized owing to their complex structure, which requires isolated middle‐lattice, strip‐like electrodes. Furthermore, polling throughout large‐scale arrays is sluggish, especially in the case of multitouch applications. With respect to commercial products, the fastest addressing speed remains lower than 8 kHz (e.g., in the case of the 3M Touch Systems in PCT*XX*). This means that polling through 1 million pixels (or a square tactile platform with a resolution of 1 K) would take 125 s, which is unacceptably long for real‐time mapping. Although an alternative method called the “Row + Column” strategy,^[^ ^3^, ^9^, ^21^ ^]^ in which all the *x*‐ and *y*‐coordinates are recorded at once to allow for 2D mapping was proposed, it resulted in “diagnose confusion” and failed to identify the locations between the reverse coordinates. This failure has limited its applicability in various areas, such as industrial control and aerospace technology.

Here, we report a high‐resolution tactile sensing array based on an arch‐bridge architecture (ABA), which is composed of an all‐transparent antifreezing hydrogel‐polymer material system. Its resistance‐based touching principle allows for crosstalk‐free output and strain‐insensitive feedback. In addition, facilitating multitouch sensing, its structure is simple, consisting only of laminations with a single inner line through each row. In particular, the electrical mode and power supply of the array have been designed to ensure that the device exhibits robust touch scanning characteristics that are not affected by interference from the flaws arising during large‐scale sensor‐to‐sensor manufacturing. Moreover, based on its ABA structure, the device allows for parallel‐addressing logic and procedures, which substantially reduce the time consumed from *n* ^2^ (for a matrix with N × N pixels) to *n*, that is, to half of that for the “Row + Column” (2*n*) approach without “diagnose confusion.” To the best of our knowledge, this is the lowest time consumed reported so far (for comparisons with other similar recently reported high‐performance devices, see **Table** **1**). It should also be noted that, owing to the structure of the device, its addressing algorithm simultaneously allows for multitouch scanning and pressure detection. The strategy proposed here should aid the development of high‐density, spatially wide tactile sensors. In addition, the simple design of the ABA should lead to the development and commercial production of systems based on different materials.

**Table 1:** Comparison of *N* × *N* ABA array with other similar previously reported high‐performance tactile sensors based on three factors—resolution, number of addressing IP (the serial number of dots or pixelated serial number) needed for polling throughout sensor (one per refresh rate of touchpad), and number of supporting lines interconnected between each dot (indicative of reliability)

*N* × *N*‐sized sensor	Resolution in terms of dots [dpi]	Numbers of addressing IP (dots) needed for polling throughout sensor	Average number of supporting lines interconnected between each dot	Sensing principle
Ref. [12]	5	2*N*	2	Triboelectricity
Ref. [13]	5.8	2*N*	2	Triboelectricity
Ref. [15]	7.4	*N* ^2^	3	Transistor resistance
Ref. [16]	5.46	*N* ^2^	3	Transistor resistance
Ref. [17]	12.5	*N* ^2^	3	Transistor resistance
Ref. [18]	5	*N* ^2^	3	Transistor resistance
Ref. [19]	5	*N* ^2^	2	Capacitance
Ref. [20]	2.5–5	*N* ^2^	2	Capacitance
Ref. [21]	10	*N* ^2^	2	Capacitance
Ref. [22]	6.25	*N* ^2^	2	Capacitance
Ref. [23]	2.5	*N* ^2^	2	Capacitance
This work	25	*N*	1	ABA strategy

## 2 Results

**Figure 1:**
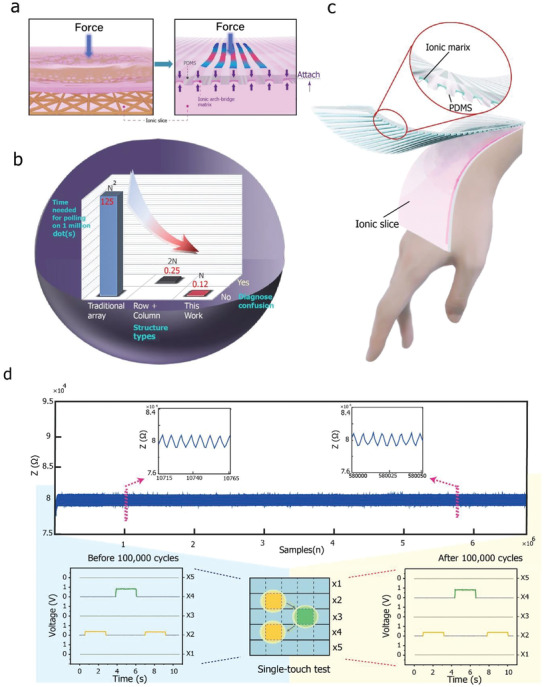
General structure and schematic illustration of antifreezing and optical properties of the proposed sensor. a) Comparison of (left) previously reported architecture and (right) ABA under the force applied. b) Comparison of scanning times. c) Illustration of ABA structure. d) During 100 000 cycles of mechanical test (stretched to 110%) along the direction of build‐in hydrogel bar, the electrical resistance records fluctuate at the constant level during the entire back‐forth process (upper part). Thus, electrical outputs corresponding to 2D position sensing before (left lower) and after (right lower) the 100 000 cycles of the mechanical test remain similar.

The proposed ABA sensor consists of only two functional laminations, namely, a suspended ionic array attached to the arch‐bridge‐shaped concave grooves formed in the upper polymer‐based layer. The lower layer consists of a slice of an ionic sheet with two strip‐like electrodes at each end. Owing to the strictly limited height of the arch bridge and the filling arrays, small gaps are formed between the upper ionic array and the lower ionic sheet to accommodate the deformation resulting from pressing. This design eliminates the need for the delicate and fragile separation net (or interseparation dots) that are commonly used in conventional touchpads (**Figure** **1**a, left), making the proposed device more durable during back‐and‐forth bending and stretching and resulting in a potentially longer lifetime. In particular, after 100 000 mechanical stretching cycles (elongation of up to 110%; resistance data were recorded during the entire cycling process), the sensor exhibited the same feedback characteristics (Figure 1d). In addition, the structure of the sensor also helps reduce light scattering between the layers to improve transparency and subsequently facilitate all‐in‐one manufacturing and assembly.

As mentioned above, under touch/force, one or more of the suspended bars move down to come in contact with the lower layer and generate touching information (i.e., voltage and current signals). In the case of resistance‐based touch sensing, the position information (i.e., the voltage output) generally depends on the homogeneity of the resistant material used. In this case, a power supply is placed at both ends of the lower ionic sheet to ensure a narrow voltage distribution along the single axis. In this manner, we can ensure that the identification of the location is accurate, because the under slice is more homogeneous than the individual suspended bar as it is much narrower. This also facilitates large‐scale manufacturing, as it removes the requirement that the suspended bars be homogeneous. Using the fabricated sensor with the ABA, the number of addressing lines can be reduced from *n × n* to *n* terminals, which can be transformed into identical *n × n* pixels in a logic matrix. Hence, the speed required for scanning every pixel and refreshing once is increased significantly based on the new addressing algorithm (Figure 1b).

An all‐transparent material, namely, polydimethylsiloxane (PDMS), and an antifreezing alginate‐polyacrylamide hydrogel (APH) with a mixed glycerin–water solvent with a volume ratio of 2:3 were chosen for the assembly of the sensor. The use of a glycerin–water phase instead of water alone can significantly improve the temperature tolerance range, which is a concern in the case of hydrogel‐based devices.^[^ ^25^, ^26^ ^]^ Inspired by LiCoO_2_/Li batteries and the use of polyaniline–polyvinyl alcohol, antifreezing organogels have been synthesized recently.^[^ ^26^ ^]^ However, flexible sensors that can operate at low temperatures remain limited in scope, and their specific assembly and manufacturing technology need to be developed further (see Figures S2 and S7, Supporting Information for a detailed discussion of the anti‐icing APH material used). In recent years, there have been significant improvements in the adhesion between ionic materials and hydrophobic substrates. However, the previously reported strategies for ensuring good hydrogel–PDMS adhesion did not work well in this case^[^ ^27^, ^28^, ^29^, ^30^ ^]^ possibly because of the oil phase present, which quenched the reaction required for good adhesion. In Figure S4 (Supporting Information), we show the fabrication processes for the APH antifreezing hydrogel and PDMS, while the results of the interfacial toughness test performed to evaluate the adhesion are shown in Figure S3 (Supporting Information). The ABA was prepared by casting the APH pregel onto a PDMS film demolded from an aluminum alloy die with a consistent number of grooves. To achieve a high density of the suspended bars in the ABA (up to ≈25 dpi, see Figure S1, Supporting Information) on a large scale, it was necessary to ensure strong and reliable adhesion between the glycerol‐containing APH and PDMS. We developed a new approach specifically for this type of interface, which involved using acrylamide as a bridge between APH and PDMS. The sensors were fabricated using the following steps: first, a pretreatment of the demolded PDMS film was performed by filling the grooves with an ethanol solution of benzophenone. This was followed by drying with N_2_ gas and immersion in an acrylamide solution, as indicated by the red arrow in Figure S4 (Supporting Information). To prevent the APH from clinging to undesired places, it is important to keep the level of the benzophenone solution lower than the protruding surface of the PDMS film and allow it to flow only along the inside of its reserved grooves. Next, APH was poured onto the pretreated PDMS film, and an aluminum alloy die with shallower grooves was used to squeeze the extra hydrogel out of the PDMS grooves to keep the height of the ionic strips uniform below the PDMS folding units. After ultraviolet (UV) curing for approximately 1 h, the hydrogel strips and their encapsulations were firmly assembled. To make the ABA more visually perceptible, the APH in the as‐fabricated sensor, which had a resolution of 6.25 dpi, was dyed using red‐violet food coloring, as shown in Figure S4 (Supporting Information) (see Figure S1, Supporting Information for photographs of the APH at other resolutions). The height of the curing gel was highly uniform, and the rest of the film remained clean and clear. Then, two platinum spring‐like electrodes were connected to the two ends of the ionic film and a 200 µm thick PDMS film was attached as the back cover. This completed the fabrication of the ABA‐based multitouch sensor. Additional information regarding the fabrication process is provided in the Experimental section. We believe that the ABA manufacturing process will also be suitable for use with other multiphase ionic material systems and high‐resolution devices.

Although the sensor was assembled using highly transparent materials, the transmittance of the free‐standing tactile matrix was found to be only 85% based on its transmission spectrum. This was owing to light scattering by the air trapped between the layers. Hence, to improve its transparency, silicon oil, which has a refractive index (approximately 1.4) similar to that of PDMS and hydrogels in general, was used as a substitute for air. Thus, in this manner, the light transparency in the visible region could be increased to 95%, as shown in blue in Figure S4 (Supporting Information).

**Figure 2:**
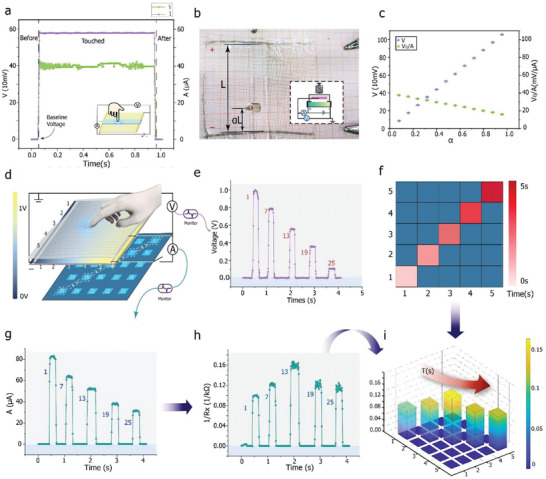
Electrical principles and properties of ABA sensor. a) Both voltage and current are recorded under same touch using fast digital multimeter. Voltage and current rise with the increase in force applied when the threshold force size of the sensor is exceeded and return to baseline values when touch is removed. b) Illustration of 1D test; inset shows electrical connection on 1D touch strip. c) Statistical voltage and current data presented as mean (points) and error (bars). Data were measured at the touch period, *α*, moving from negative to positive end. d) Schematic of 2D touch sensor with five channels of ABA output in 5 × 5 logic matrix. e) Recorded output voltage sequence during 2D test at five marked positions along diagonal. f) Output of 2D coordinates as interpreted from voltage signals and serial number in ABA channels. g) Current signal recorded during the above‐described touching process. h) Force specified by the relationship between current *A*, and *α*. i) 3D diagram for entire process showing 2D coordinates and Force together.

The structure and measurement circuit for a cross‐section of the device during a 1D test are shown in the insets of **Figure** **2**a,b; only a single iontronic suspended bar is connected to the system. A source voltage was connected to the two spring‐like platinum electrodes. As per the rules of voltage division, the voltage drop in the long platinum electrodes can be ignored because the ratio of the resistance of the hydrogel slice and the impedance of the interface between the metallic and hydrogel layers was lower than 1:1000. When force is applied between the two electrodes, the voltage drop occurs strictly along the side perpendicular to the spring electrodes, as recorded by a voltmeter connected to the ionic channels of the ABA structure. In addition, to determine the relevant load size, an ammeter was connected between the ABA structure and the positive end. The normalized distance is represented by *α*, which indicates the touch position on the touchpad, as shown in Figure 2b. When the 1D strip is touched by a finger, the voltage and current signals generated can be represented by the following equations:

(1) $$V\propto \frac{\alpha L}{L}\times \frac{R_{0}}{R_{0}+2Z_{\mathrm{EDL}}}\times V_{0}=\alpha V^{\prime }$$

(2) $$A=\frac{V^{\prime }}{\alpha R_{1}+\left(1-\alpha \right)R_{0}+R_{x}}$$

where *R_0_, R_1_*, and *R_x_* are the resistances of the ionic even‐surface layer, individual ABA, and pressure resistance (PR), which is caused by the impedance of the electrical double layer, which varies with the applied force. *V*_0_ refers to the original power applied to the sensor, and *V*′ represents the effective voltage drop on the ionic even surface, excluding the contact impedance between the wires and the APH. Notably, the pressure, which can be physically represented as PR, is correlated with both the current value and the touching position. On the other hand, *α* is only related to the voltage signal.

During the 1D test, the latency of the sensor was ≤ 10 ms (Figure 2a) during rapid finger touching. Figure 2b shows a load of 500 g applied to 12 touching positions normalized from zero to one along the strip. The voltage was recorded under a specific pressure, as shown in purple in Figure 2c. It can be seen clearly that the voltage was linearly proportional to the distance of the touching position from the ground terminal (GND); this was in keeping with the mathematical expression in Equation (1). In addition, electrical current signals were also recorded, as marked in green in Figure 2c. It can be seen that the current generated was inversely proportional to *α* unless the applied pressure was changed.

A 2D matrix was tested using the same measurement circuits with a 5 × 5 matrix. Figure 2d shows that the color map of the ionic even‐surface slice changes from blue to yellow based on the distribution of the voltage supplied by the source voltage, *V*′. Five ABA channels on the upper layer were chosen for testing and connected to a data acquisition (DAQ) multimeter. To allow for better visualization, the 5 × 5 pixels were labeled in sequence from 1 to 25 on the map, and the 2D test was performed along the diagonal track. The data acquired synchronously at the five ionic channels, that is, the voltage and current signals, are shown in Figure 2e,g, respectively. The conversion of the voltage data into the 2D coordinates was normalized as follows:

(3) $$\left(\alpha ,\beta \right)=\left(\left[\frac{1}{0.2}\times \frac{n_{i}}{N}+1\right],\left[\frac{1}{0.2}\times \frac{V}{V^{\prime }}+1\right]\right)$$

In this equation, the total number of channel arrays is assumed to be *N*, with the serial number being *n_i_*. The collected voltage is *V*, while *V’* is the total voltage difference between the two strip‐like electrodes on the other part of the sensor. As the final outputs are 2D‐position should be an integer, the result is rounded to make it an integer between one and five. The result of the 2D coordinate projection obtained by this strategy is shown in Figure 2f; the touching sequence is labeled as a red square whose color varies from a shallower hue to a deeper one. Finally, a 3D diagram that combines the coordinates and force information (see Figure 2h) is shown in Figure 2i; in this figure, the *z*‐axis represents the applied pressure.

**Figure 3:**
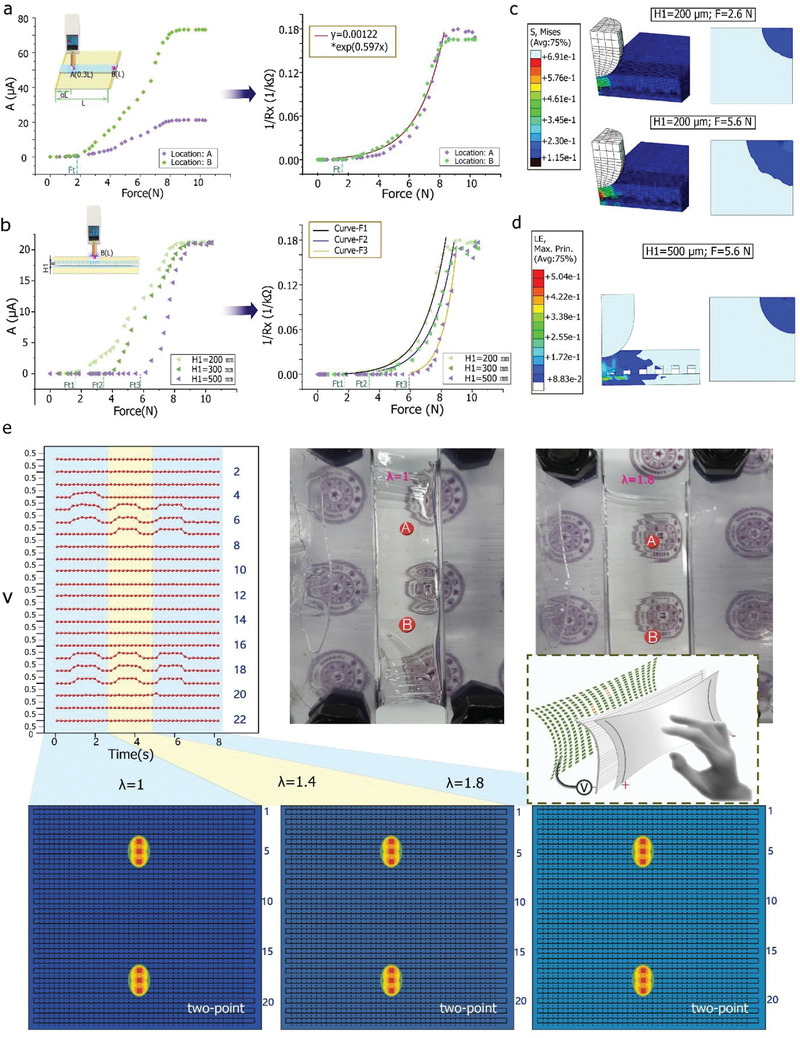
Results of pressure and anti‐interference measurements during operation of the flexible device. a) For point *A* to *B*, (left) curve of current in ABA sensor against pressure and (right) converted force value. b) Force–current curves for *H*1 values of 200, 300, and 500 µm and corresponding fitting curves. Curve for F1 function is “*y* = 0.00063*1.997*^x^*”; that for F2 function is “*y* = 0.00033*2.23*^x^*”; and that for F3 function is “*y* = 0.00000089*3.9*^x^*”; c) Sectional and top‐view images obtained during finite‐element method (FEM) simulation showing deformation distribution of finger at *H*1 = 200 µm. d) Images obtained during FEM simulation at *H*1 = 500 µm. e) Voltage output at multitouch positions *A* and *B* on 2D 22‐channel touchpad as recorded thrice using multiplex digital‐to‐analog converter during stretching process from elongation *λ* = 1 to *λ* = 1.8.

The exact value of the applied force can be obtained by integrating the current data with the corresponding coordinates (**Figure** **3**a–d). The current signal as measured by the digital force meter is positively related to the applied force, which can also calculate by the relationship between the contact resistance *R_x_*, and the force value (see Equation 2). During this experiment, the applied force monitoring by the digital display force meters with a ball head at the tip poke at points *A* and *B* concurrently, and the corresponding current data are highlighted in Figure 3a in purple and green, respectively. The results indicate that, regardless of the position of touch, the physical relationship between the contact resistance *R_x_*, arising from the interface of the conductive layers and the force value can be fitted using the following equation:

(4) $$y=\frac{1}{R_{x}}=0.00122\times e^{0.597x}$$

This exponential curve can be further fitted to a linear one as follows:

(5) $$y^{\prime }=\ln y=-\ln R_{x}=0.00122+0.597x$$

To elucidate the mechanism of the inner deformation process and signal transduction, we simulated the procedure using the finite element method (FEM) (Movie S4, Supporting Information). The cross‐sectional (Figure 3c, left) and top view (Figure 3c, right) were simulated for forces of 2.6 (Figure 3c, up) and 5.6 N (Figure 3c, down). The figures show that once the applied force exceeds the threshold *F*_t_, current is generated on the ABA strips through the touchpoint from the positive end to the negative one, with the total electrical resistance being (1*‐α*)*R*_0_ + *αR*_1_ + *R_x_*, where *R_x_* is related to the degree of compaction of the conductive interface and its contact area. The cross‐sectional FEM analyses confirmed that inner deformation occurs under pressure, resulting in the tightening of each layer, while the top‐section analyses showed the increase in the size of the contact area.

To investigate the effect of the gap (*H*1) between the iontronic suspended bar and the bottom APH electrode on *F*_t_ and the force sensitivity, *H*1 values of 200, 300, and 500 µm (Figure 3b,d) were selected to elucidate the influence of the above‐mentioned relationships. Figure 3b shows that the variations in the height directly affected the threshold pressure, *F*_t_, and the slope of the curve for the function but only slightly affected its mathematical expressions. When the height was varied from 200 to 500 µm, the function for the relationship between *y* = *1/R_x_* and the pressure value *x*, could be fitted as follows:

(6) $$H1:\;y=0.00063\times 1.997^{x}$$

(7) y'=lny=ln0.00063+xln1.997

(8) $$H2:y=0.00033\times 2.23^{x}$$

(9) $$y^{\prime }=\ln y=\ln0.00033+x\ln2.23$$

(10) $$H3:\;y=0.00000089\times 3.9^{x}$$

(11) y'=lny=ln0.00000089+xln3.9

The FEA simulation results obtained at *H*1 = 500 µm are shown in Movie S5 (Supporting Information), while an image captured during the simulation at 5.6 N is shown in Figure 3d. It can be seen that the upper suspended bar structure has not yet come in contact with the bottom APH layer at a force of 5.6 N. The threshold pressure *F*_t_, values obtained experimentally and those obtained through the simulations under the same conditions were similar and increased with the increase in *H*1.

The anti‐interference performance of the device during back‐and‐forth stretching was evaluated using a sensor with 22 × 22 logic pixels (Figure 3e). The upper left part of Figure 3e shows the 22‐channel data recorded during stretching from *λ* = 1 (initial, the upper middle part in Figure 3e) to *λ* = 1.8 (80% prolonged stretching, upper right part in Figure 3e) with the finger touching markers *A* and *B*. The corresponding positional information obtained from the voltage data is given in the lower panels in Figure 3e, which show that the position recognition data for *λ* = 1, 1.4, and 1.8 are highly uniform and consistent with each other. Therefore, it can be concluded that the antistretching ability of the device is robust and not affected by flexible motions such as stretching and bending.

**Figure 4:**
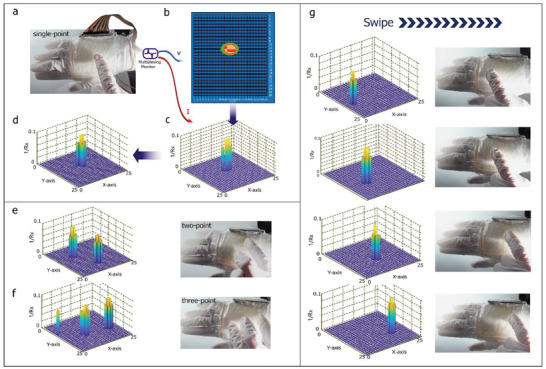
Single‐point touch, multipoint touch, and swipe detection results. a) Schematic of as‐fabricated 25‐channel sensor connected to DAQ multimodel system for gesture recognition test. b) Voltage recorded and its conversion to 2D coordinates. c) 3D bar displaying integration information with pressure. d) Final result obtained using an edge‐compensation algorithm. e–g) Outcomes for two‐point touch, three‐point touch, and single swipe detection, respectively.

In addition, the sensor can detect various types of gestures and commands, ranging from a single touch to a three‐point touch and from a single swipe to multiple swipes. This was confirmed using a sensor array of 25 × 25 pixels in a 25 cm^2^ logic matrix. The real‐time feedback of the 2D coordinates and force (*1/R_x_*) was recorded and processed using a software based on the 25 channel multiplexing data models with voltage and current monitoring (**Figure** **4**a). The results of the single‐point touch test are shown in Figure 4b,c. The bar diagram in Figure 4c also shows information regarding the diameter of the fingerprint across three and four pixels in this case. To accurately mimic the round trail of the finger touch and compensate for the resulting cut in the shape, a cluster of round bars is provided using a specific algorithm called the “edge‐compensation” program, which is based on the knowledge of the recorded diameter (Figure 4d). Additional gesture recognition results are given in Figure 4e–g, where the 3D bars clearly show a multipoint touch and a single swipe.

**Figure 5:**
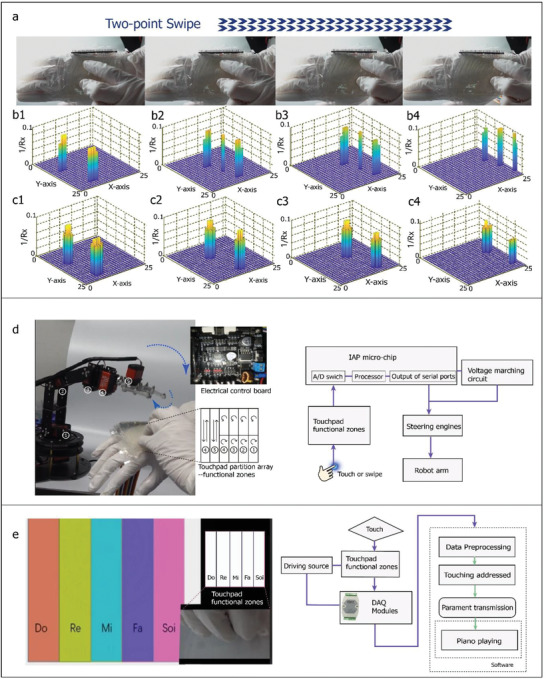
Two‐point swiping, controlling of robot arm, and piano playing. a) Photographs showing a procedure for two‐point swiping. b) Initial output of swiping process with additional “third” point sequentially numbered as 2 to 4. c) Final output obtained using the enhanced edge‐based program. d) Controlling robot arm. e) Playing piano computer games; configuration of the electrical system is shown on the right side.

Next, the performance of the device was evaluated during a two‐point swipe under exceptional conditions, wherein we deliberately kept two fingers vertically on the ABA sensors such that the two points were located on the same iontronic suspended bar in the ABA structure (**Figure** **5**a). Because of the high resolution of the ABA, the sensor could still recognize the two points, as shown in Figure 5b1. However, it also reported a “third” point between the actual ones, as shown in Figure 5b, owing to the fact that different voltages were connected to the same channel, resulting in the formation of a small local current loop between the touch positions. Hence, the average value of the voltage was determined mathematically and output by the software. Taking advantage of the high resolution of the ABA, we developed an enhanced detection program based on a previous finger‐edge detection algorithm to ensure that the device performs as desired even under extreme conditions. Once the algorithm is running, the unwanted position and its flagged voltage were removed, and the final outcome was determined based on the edge‐detection information and compensation was performed based on the diameter of the fingerprint to form a round touch print (Figure 5c).

Next, we determined whether the sensor can be used for human–machine interfaces, such as the wearable control panel for a robotic arm (Figure 5d and Movie S2, Supporting Information) and a piano‐playing panel (Figure 5e and Movie S3, Supporting Information). Figure 5d shows that the area of the sensor is divided into 10 partitions corresponding to different commands, with six analog channels connected to a microchip (see the right side). The playing‐piano game was implemented using the above‐mentioned DAQ modules as the hardware and the LabView program in MATLAB as the software.

## 3 Conclusions

In conclusion, by using an ABA‐based sensor, we were able to realize an algorithm for parallel swiping. We developed and evaluated a high‐resolution tactile sensor and could achieve real‐time mapping at an addressing speed, that, to our knowledge, is unprecedented for large‐scale pixelated sensors. Compared with previous devices, the one fabricated in this study is significantly more compact and concise and thus scalable and suitable for mass fabrication. In addition, it should exhibit a long lifetime when subjected to various operations involving repetitive motions. The parallel‐swiping algorithm and the supporting edge‐compensation program were discussed in detail. The design of the ABA sensor will undoubtedly broaden the applicability of resistance‐based sensing and aid the development of highly robust and stretchable multitouch sensors that are simultaneously crosstalk free and show strain insensitivity while allowing for high‐resolution recognition in real time. The electrical supply used is simplified to consist of only two electrodes instead of *n* electrodes, as this prevents interference during position sensing owing to the inherent flaws that arise during sensor‐to‐sensor manufacturing. In addition, given that large‐scale units based on unified sensor platforms are generally plagued by poor reliability and sluggish feedback, we believe this study provides a robust technique for the fabrication of high‐density, large‐spatial sensors for real‐time million‐pixels processing in the future.

## 4 Experimental Section

### Details of Sensor Fabrication—Preparation of ABA Sensor

Unless otherwise stated, the resolution of the ABA sensor used in this study was 39 dpi. First, the PDMS substrates were fabricated using Sylgard 184 (Dow Corning) in a catalyst weight ratio of 10:1. The silicone base of the ABA structure was demolded from an aluminum die to form a concave–convex surface with the designed resolution. To allow for easy demolding, the alumina alloy was treated with a plasma cleaner (PDC‐001) for 1 min and immersed in perfluoro caprylic acid (Aladdin 335‐67‐1) at 50 °C for 12 h to form a hydrophobic surface. After these three steps, the APH antifreezing hydrogel adhered to the PDMS substrate in the precisely designed shape; this structure is denoted as L1.

The lower part of the sensor, which was composed of an even film of APH, was also cured under UV light and attached to the PDMS encapsulation slice (BALD silicone films Company, KRR‐200, elasticity of 0.6 MPa, and elongation at break of 900%) after the completion of the three steps mentioned above. This structure is denoted as L2. In addition, both slices were dipped into a 2 m Ca^2+^ solution (anhydrous calcium chloride, Aladdin) to improve the toughness of the hydrogel. The final sensor was assembled by attaching L1 to L2 by forming a plasma bond between the PDMS surroundings.

### Experimental Design—Readout of Electrical Properties

A direct power source (1.5 V) was used for the testing the electrical characteristics unless noted otherwise. The resistance measurements during the antifreezing test were performed with an LCR meter (UCE2831) at a frequency of 1 kHz using a piece of APH hydrogel with dimensions of 10 × 25 × 1.5 mm^3^.

Two digital desktop multimeters (Keysight 34470A and Victor 8246A) were used in the 1D test to record the voltage and current data. The 2D electrical and gesture recognition tests were performed using eight‐channel DAQs in parallel (ART Technology 3202 for the voltage signal and PXI7062A for the current signal through a PXI2601 scanning switch) to allow for propagation through the ABA channels. The acquired data were recorded and processed in real time using LabView. MATLAB was incorporated with LabView to implement the piano‐playing game. For the other application, that is, for robot control, a laboratory‐made circuit board with a microchip and an eight‐channel analog‐to‐digital converter was connected between the five steering engines and ABA sensors, which had six channels that responded to the control commands.

The mechanical cycling test was performed on a five‐channel ABA sensor using a laboratory‐made single‐axis stretching machine. The test was performed for 100 000 cycles, and the impedance data from one of the five channels served as the cycling marker, which was recorded with an LCR meter (UCE2831). Before and after the cycling test, the five‐channel ABA sensor was touched by a finger, and touch‐position sensing was performed using DAQs placed in parallel (ART Technology 3202). The test to evaluate the strain insensitivity along two directions vertical to each other (Figure S6, Supporting Information) was carried out using a 24‐channel ABA sensor. The touch positions were marked on the sensor's surface. In addition, some of the working channels were also previously selected and connected to the multiplexing models to record the output in parallel (ART Technology 3202).

### Statistical Analysis—Preprocessing of Data

The sampled data from DAQ models was smoothed by three points before any subsequent process; the sampling data of single‐axis stretch‐stress tests as the import data for Finite analysis is pre‐processed as average value in three times.

### Statistical Analysis—Data Presentation

In Figure 2c, Data were presented in the form of “mean ± SD”, with four samples at every points).

### Statistical Analysis—Sample Size(n)

The total sample size for each statistical analysis is in list below Figure 1d (up)—81857; Figure 1d (down)—2540; Figure 2a—1013; Figure 2c—48; Figure 2e—410 Figure 2g—415; Figure 3a—88; Figure 3b—132; Figure 3e—880; For Gesture detection Figures 4c–5a—1575; For Figures in Supporting Information: Figure S2d (Supporting Information)—82; Figure S3 (Supporting Information)—13490; Figure S4b (Supporting Information)—601; Figure S5a,b ( (Supporting Information)—41, 41; Figure S6ac (Supporting Information)—242, 1072; Figure S7b (Supporting Information)—100; the sampling data of single‐axis stretch‐stress tests as the import data for Finite analysis is 650 on average either for PDMS or for hydrogels.

### Statistical Analysis—Statistical Methods

In Figure 3a,b, the exponential functions are selected for curve‐fitting analysis to make less error.

Software used for statistical analysis is MATLAB & Origin.

## Conflict of Interest

The authors declare no conflict of interest.

## Supporting information

Supporting Information

Supplemental Movie 1

Supplemental Movie 2

Supplemental Movie 3

Supplemental Movie 4

Supplemental Movie 5

Supplemental Movie 6

Supplemental Movie 7

## Data Availability

Research data are not shared.
