# Supplementary material for: Crosstalk‐Free, Stretching‐Insensitive Sensor Based on Arch‐Bridge Architecture for Tactile Mapping with Parallel Addressing Strategy toward Million‐Scale‐Pixels Processing
Source: Adv Sci (Weinh). 2021 Sep 9;8(21):2101876. doi: 10.1002/advs.202101876 (PMC8564424; doi:10.1002/advs.202101876)
Supplement: Supplementary file 1 — Supporting Information [file ADVS-8-2101876-s008.pdf]

WILEY-VCH

© 2020 Wiley-VCH GmbH

Supporting Information

for *Adv. Sci.*, DOI: 10.1002/advs.202101876

Crosstalk-free, stretching-insensitive Sensor Based on arch-bridge Architecture for Tactile Mapping with Parallel Addressing Strategy Toward million-scale-pixels Processing

*Ruomei Shao, Chunnan Wang, Jingru Zhao, Hang Yang, Shuqing Sun**

© 2020 Wiley-VCH GmbH

Supporting Information

Crosstalk-free, stretching-insensitive sensor based on arch-bridge architecture for tactile mapping with parallel addressing strategy toward million-scale-pixels processing

Ruomei Shao[1,†], Chunnan Wang[1,†], Jingru Zhao[1], Hang Yang[2], Shuqing Sun[1]*

[1] Institute of Biopharmaceutical and Healthcare Engineering, Tsinghua-Shenzhen International Graduate School, Tsinghua University, Shenzhen 518055, China

*Correspondence to: sun.shuqing@sz.tsinghua.edu.cn

[2]State Key Laboratory for Strength and Vibration of Mechanical Structures, International Center for Applied Mechanics, Department of Engineering Mechanics, Xi'an Jiaotong University, Xi'an 710049, China

[†]These authors contributed equally: Ruomei Shao, Chunnan Wang.

This PDF file includes:

Supplementary Text

Figs. S1, S2, S3, S4, S5, S6, S7

Table S1

Other Supplementary Materials for this manuscript include the following:

Movies S1 to S7

Supplementary Text

The anti-icing performance for transparent double-phase hydrogels:

By using oil–water mixture solvent instead of a water phase, the anti-acing materials also demonstrates exceptional temperature tolerance down to −30 ℃ and allows operation in a wide temperature range and under various circumstances. Fig. S1 shows the cold-resistance performance relative to the gradient volume concentration of glycerin to water from 0%–50%. The ionic materials remains transparent and mechanically soft at a temperature of −40 ℃ at a volume ratio of 40%–50% (the performance in other temperature see Fig. S1a,b,c), and its electronic characteristics are shown in Fig. S1d by a volume ratio of 40%. By further increasing the ratio of the glycerin phase up to more than 40%, the toughness of the APM hydrogel can be abruptly impaired, possibly because of the high ratio of glycerin mixed in water, which hinders the cross bonding of acrylamide. By considering both the anti-freezing property and mechanical performance of the conducting layer, the ratio of glycerin–water is optimized by 40% for the sensor fabrication.

A 24-channel matrix is fabricated and preserved in a commercial deep-frozen refrigerator at -40 ℃ for 24 hours. And then the matrix is taken out for observation by eyes and characterization by LCR meters.As Figure S7 shows, under -40℃,the matrix remains flexible and the ionic bars are still conducitive. Once taken out from deep fozen surroundings, there is a thin layer of ice quickly condensed on its surface (air humidity in Shenzhen at that time is about 95%). It is found that after frozen, the resistance of the ionic bars decreased slightly and gradully recovered to original value after it is warmed up. However, the resistance along the bars are still homogenous and the sensing properties of the device remains the same.

The outcome of toughness interface bonding test is displayed in Figure S3. The test is carried out as 180° peeling-force applied by a universal testing machine. Its result clearly shows that compared with the former reported approaches the bonding strength of glycerin-existed interface between APH and PDMS is largely improved by 10 times around. The peeling test is performed using samples with 100 mm in length, 18 mm in width and 2 mm in thickness. The interfacial toughness is calculated by dividing the steady-state peeing force with the sample width.

FEM Simulation of ABA deformation under finger-mimic press:

To elucidate the underlying mechanism between contact resistance and pressure visually, we further establish a 3D sensor model with material systems in ABAQUS, varying the height of H1 at 200 and 500 µm. The models of sensor's structure are established in a subarea of 1/4 for a minimum computation. Due to the hyperelasticity property in the materials, our sensor can be modeled as an integrated platform with Arruda—Boyce hyperelastic characteristics. Here, for a small deformation relatively, the Arruda—Boyce model is used without a correction term to define its distortion boundary. The model equation in ABAQUS can be expressed as:

$$U = \mu \sum_{i=1}^{5} \frac{C_i}{\lambda^{2i-2}} (\mathrm{I}_1^i - 3^i) + \frac{1}{D} \left(\frac{J_{el}^2 - 1}{2} - \ln(\mathrm{J}_{el}) \right) \qquad \text{(S1)}$$

where U represents the energy potential, C_1= 1/2, C_2= 1/20, C_3= 11/1050, C_4= 19/7000, and C_5= 519/673750, I_1 refers to Partial strain, and J_{e1} is the volume ratio. µ and λ can be calculated by measured stress-strain data and stand for the Arruda—Boyce parameter, represents a series of principal stretches. D is a constant related to the bulk modulus of elasticity in which D=0 means an incompressible property.

By the Curve Fitting routine in ABAQUS, the uniaxial test data as mean to fit it into the Arruda—Boyce model is displayed in Supplementary Fig. S5. Meanwhile the output of fitted parameters is listed in Supplementary table S1, where μ_0 is represented as the consistent shear modulus of elasticity.

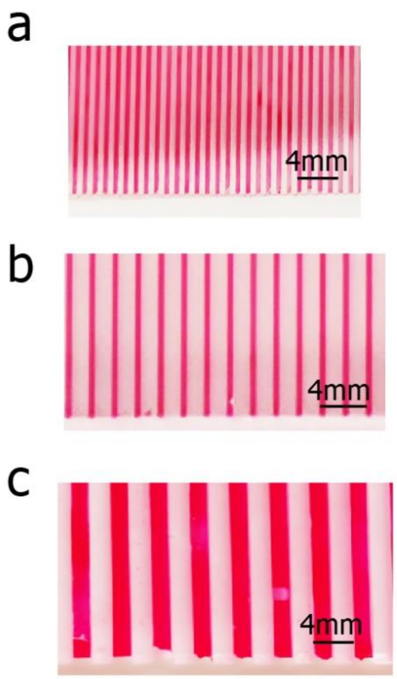

Figure S1 Three different density on ABA matrix with APH hydrogels dying in red. a) 25dpi; b) 12.5dpi; c) 6.25dpi.

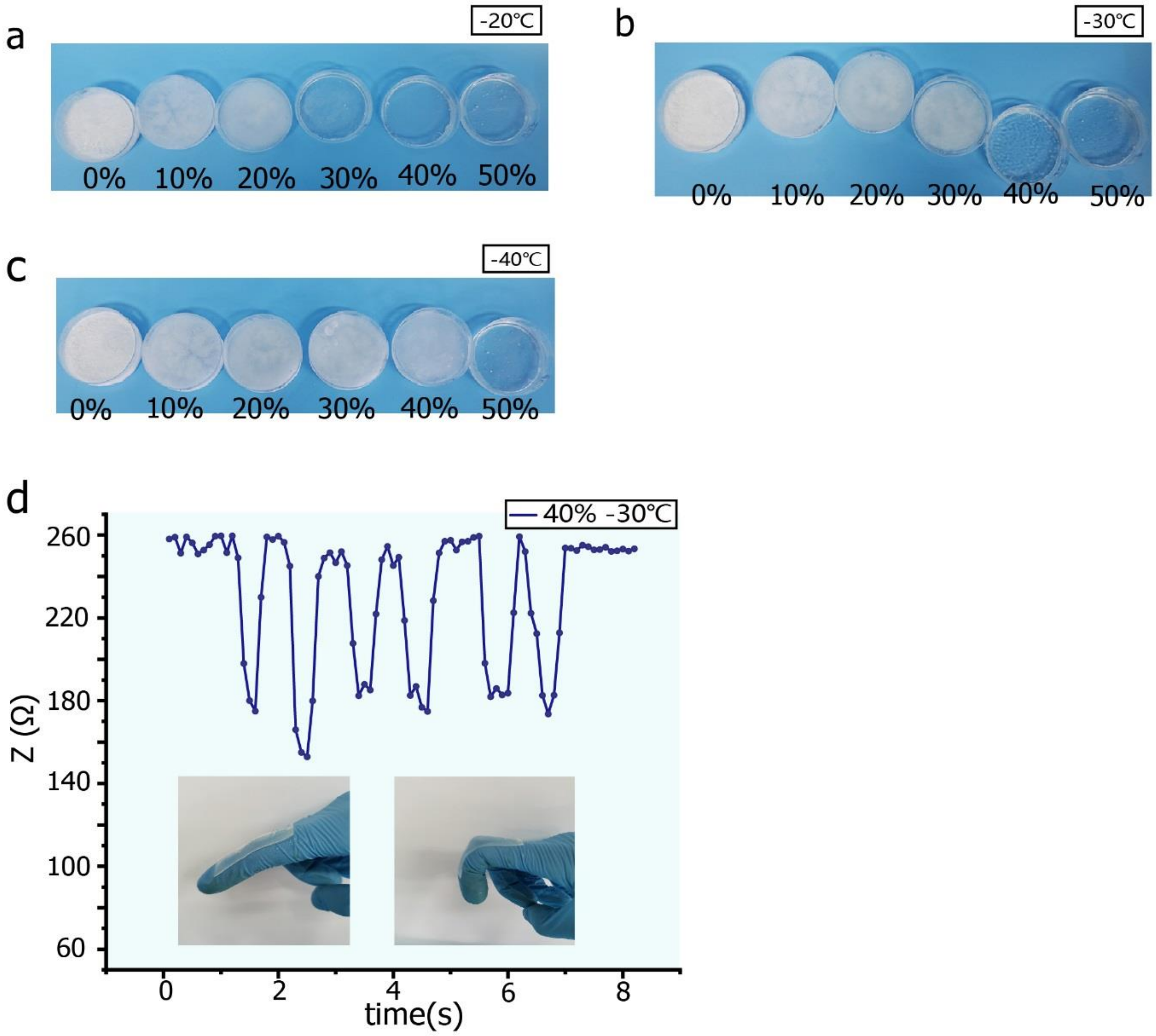

Figure S2 Anti-freezing test of ionic conductor with concentration gradient of glycerol from 0% to 50% shown in the pictures. a) the test under -20℃; b) under -30℃; c) under -40℃; d) flexible test of hydrogels at −40 °C with electrical output.

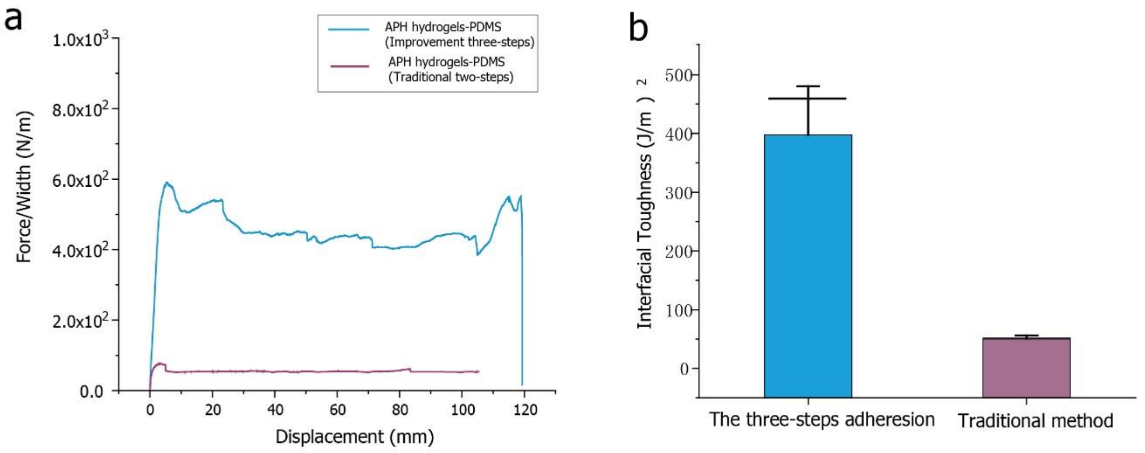

Figure S3 Interfacial toughness for as-prepared anti-freezing hydrogels attached on PDMS substrates with different methods. **a**) Typical curves of peeling force per hydrogel width vs. displacement; **b**) The measured interfacial toughness for various as-prepared anti-freezing hydrogels to PDMS substrates (n=4~5).

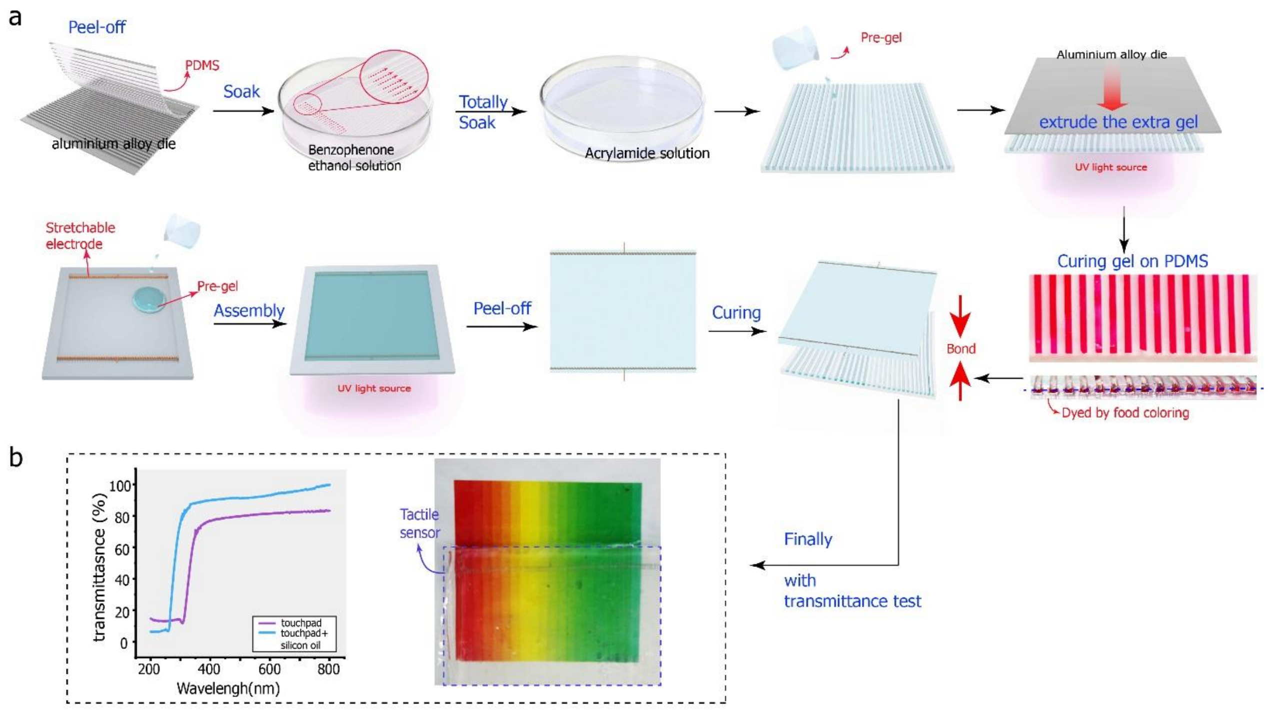

Figure S4 a) Fabrication process. b) Transmission spectra of ABA sensors (left) with and (blue) without a refractive matching ingredient. Optical image of the transparent ABA sensors on top of the color card.

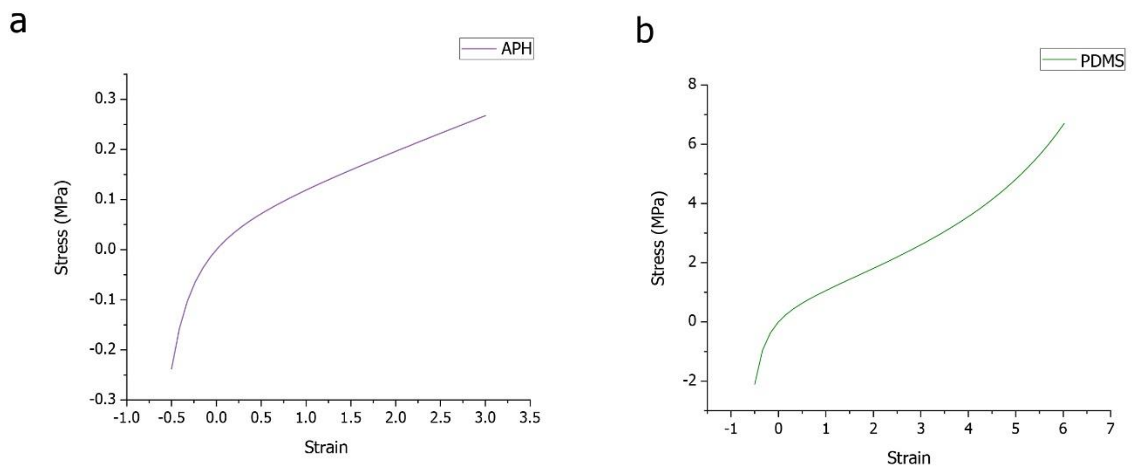

Figure S5 Statics meanly strain-stress curves on uniaxial test: a) APH; b) PDMS.

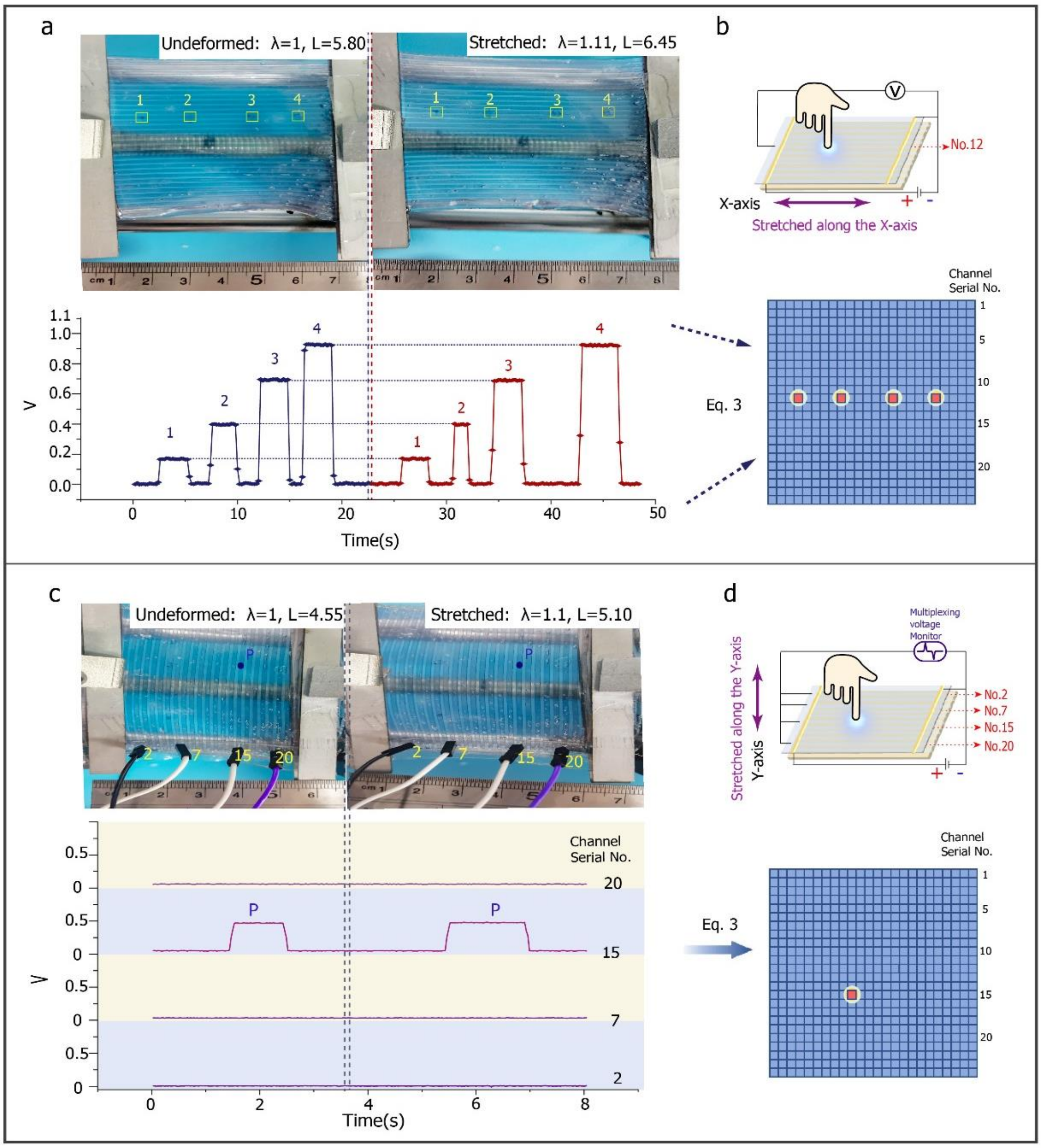

Figure S6 Strain-insensitive performance tests on position-sensing along both directions of X-axis and Y-axis. Specially, 24-channels touch sensor is fabricated by the above-mentioned process, with its ionic parts is dying in deep blue by food coloring—to make the direction clearer. Notably, the experiment is started off with touch-position marked on the sensors by mark pens. a, c) The optical images of stretched sensor along Y-axis with marked positions (up); Voltage outputs before and after stretching along the Y-axis (a)/ X-axis (c) to 10% strain (down); b, d) Schematics of the stretching test on Y-axis (b)/ X-axis (d).

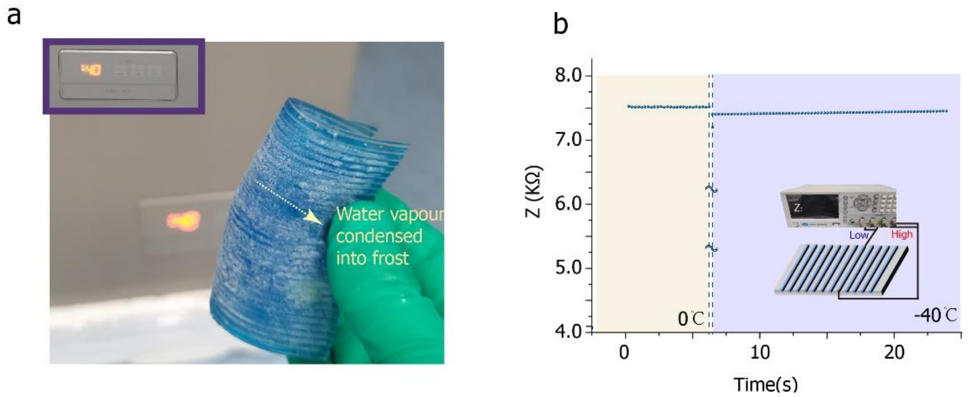

Figure S7 -40℃ frozen test on 24-channel matrix. Dying with deep blue, a 24-channel matrix is fabrication and preserved in a commercial deep-frozen refrigerator at -40℃ for 24 hours. And then the matrix is taken out for observation by eyes and LCR meters. a) The optical images for just taken out matrix; **b)** Conductivity check and comparison before and after deep frozen.

Table S1

Parameters	μ	μ_0	λ	D
APH	6.790901134×10^{-2}	6.790901196×10^{-2}	8090.33384	0
PDMS	0.584404806	0.596262987	5.52407902	0

Movie S1

Different gesture recognition on single-touch, multi-touch, single-strip, multi-strip.

Movie S2

A ABA touch pad is served as human-computer interfaces on playing computer piano games.

Movie S3

A ABA touch pad is served as human-robot arm interfaces to deliver different controlling commands.

Movie S4

The sectional view of FEM simulation to elucidate the inner deformation of sensors with the 200 µm height of H1.

Movie S5

The top-view of FEM simulation to elucidate the inner deformation of sensors with the 200 µm height of H1.

Movie S6

The sectional view of FEM simulation to elucidate the inner deformation of sensors with the 500 µm height of H1.

Movie S7

The top-view of FEM simulation to elucidate the inner deformation of sensors with the 500 µm height of H1.
